# Exploring rare and low-frequency variants in the Saguenay–Lac-Saint-Jean population identified genes associated with asthma and allergy traits

**DOI:** 10.1038/s41431-018-0266-4

**Published:** 2018-09-11

**Authors:** Andréanne Morin, Anne-Marie Madore, Tony Kwan, Maria Ban, Jukka Partanen, Lars Rönnblom, Ann-Christine Syvänen, Stephen Sawcer, Hendrik Stunnenberg, Mark Lathrop, Tomi Pastinen, Catherine Laprise

**Affiliations:** 1 Department of Human Genetics, McGill University, Montréal, QC Canada; 2 McGill University and Genome Québec Innovation Centre, Montréal, QC Canada; 3 Département des Sciences Fondamentales, Université du Québec à Chicoutimi, Saguenay, QC Canada; 4 Department of Clinical Neurosciences, University of Cambridge, Cambridge, UK; 5 Research & Development, Finnish Red Cross Blood Service, Helsinki, Finland; 6 Department of Medical Sciences, Section of Rheumatology, Uppsala University, Uppsala, Sweden; 7 Department of Medical Sciences, Molecular Medicine and Science for Life Laboratory, Uppsala University, Uppsala, Sweden; 8 Department of Molecular Biology, Faculty of Science, Radboud University, Nijmegen, The Netherlands; 9 Center for Pediatric Genomic Medicine, Kansas City, MO USA; 10 Centre Intégré Universitaire de Santé et de Services Sociaux du Saguenay–Lac-Saint-Jean, Saguenay, QC Canada

**Keywords:** Genomics, Epigenetics, Genomics, Epigenetics

## Abstract

The Saguenay–Lac-Saint-Jean (SLSJ) region is located in northeastern Quebec and is known for its unique demographic history and founder effect. As founder populations are enriched with population-specific variants, we characterized the variants distribution in SLSJ and compared it with four European populations (Finnish, Sweden, United Kingdom and France), of which the Finnish population is another founder population. Targeted sequencing of the coding and non-coding immune regulatory regions of the SLSJ asthma familial cohort and the four European populations were performed. Rare and low-frequency coding and non-coding regulatory variants identified in the SLSJ population were then investigated for variant- and gene-level associations with asthma and allergy-related traits (eosinophil percentage, immunoglobulin (Ig) E levels and lung function). Our data showed that (1) rare or deleterious variants were not enriched in the two founder populations as compared with the three non-founder European populations; (2) a larger proportion of founder population-specific variants occurred with higher frequencies; and (3) low-frequency variants appeared to be more deleterious. Furthermore, a rare variant, rs1386931, located in the 3ʹ-UTR of *CXCR6* and intron of *FYCO1* was found to be associated with eosinophil percentage. Gene-based analyses identified *NRP2, MRPL44* and *SERPINE2* to be associated with various asthma and allergy-related traits. Our study demonstrated the usefulness of using a founder population to identify new genes associated with asthma and allergy-related traits; thus better understand the genes and pathways implicated in pathophysiology.

## Introduction

The Saguenay–Lac-Saint-Jean (SLSJ) region is located in northeastern Quebec and is known for its unique demographic history and founder effect, characterized by several population bottlenecks followed by rapid expansion [1]. Founder populations, like SLSJ, are usually less diverse at the environmental and genetic levels [2], and harbor larger identical DNA segment than outbred populations [3, 4]. An advantage of studying founder populations is the possibility to investigate variants, which occurred in higher frequencies as compared with the general population, due to either genetic drift or weaker selection against deleterious alleles [5–8]. These effects can lead to overcome selection disadvantage and allow alleles to reach higher frequencies in founder populations [9–12] such as the French-Canadian [13] and Finnish populations [14]. These characteristics make founder populations well suited to study the impact of rare and low-frequency variants in complex traits, such as asthma [11, 15, 16]. Recent studies showed the smaller but important contribution of rare and low-frequency variants in the genetic basis of complex traits [17, 18]. Despite the major contribution of common variants, investigations of rare and low-frequency variants in founder populations would provide additional and insightful information about the underlying biological mechanism of traits development.

Rare and low-frequency variants have been previously studied to better understand the genetic basis of complex traits such as asthma and allergic diseases [19–21]. Although these variants do not explain a large part of the missing heritability they contribute nevertheless [19]. Rare variants exploration identified previously associated genes (ex: *GSDMB* [13], *IL33* [15]) and new ones (ex: *GRASP* [13]), highlighting the importance of studying their role in the context of asthma and allergy-related traits.

In this study, we set out to identify new variants and genes associated with various traits related to asthma and allergy in hope to better understand the biological mechanisms and pathways of these diseases. We aimed to take an advantage of the well-characterized SLSJ cohort and its availabilities of comprehensive phenotypes related to asthma and allergy [1]. Our first objective is to characterize the variants distribution in the SLSJ founder population and to compare it with four European populations (i.e., Finland (FINN), Sweden (SWE), United Kingdom (UK) and France (FR)). Of the four populations, the Finnish is another founder population. As SLSJ is part of the French-Canadian population, we wanted to follow-up on previous results and see if we could observe the same enrichment of deleterious variants in this population [13]. Our second objective is to explore the functional consequences of identified rare and low-frequency variants and their respective genes in asthma and allergy-related traits in the SLSJ asthma familial cohort [1]. Given that asthma and allergies are characterized by multiple subphenotypes and endotypes, we selected the quantitative traits related to both the immunological and clinical aspects for investigations: serum immunoglobulin (Ig) E levels, eosinophil percentage and lung function.

## Materials and methods

### Samples

We sequenced samples from 149 parent–child trios (447 samples) from the SLSJ asthma familial cohort [1]. For the characterization of variants distribution, of the 447 samples, 93 non-related parents were selected and paired by mean coverage to avoid bias with 93 non-related samples from each of the four study populations (FINN, FR, SWE and UK) for a total of 465 samples. All studies received ethic approbations from their respective ethic committees.

To analyze the impact of rare variants on the phenotypes of lung function (i.e., forced vital capacity (FVC), forced expiratory volume in 1 s (FEV_1_) and Tiffeneau–Pinelli index (FEV_1_/FVC)), serum IgE levels and eosinophil percentage, the sequenced data from the 149 sequenced trios were used to infer and impute genotypes for the rest of the cohort (1214 individuals from 254 families [1]; see *Inference and imputation* section; see Table 1 for Clinical description and for Recruitment details). Lung function, serum IgE levels and differential white blood cell counts of the SLSJ cohort have been measured and published previously [1]. The study was approved by the *Centre Intégré Universitaire de Santé et de Services Sociaux du SLSJ* ethics committee. All subjects gave informed consent.

**Table 1:** Clinical description of the SLSJ asthma familial cohort

	All samples (*n* = 1214)	All trios (*n* = 447)	Probands^a^ (*n* = 149)	Parents (*n* = 298)	Siblings (*n* = 110)
General characteristics					
M:F ratio	1 :1.2	1:1.1	1:1.3	1:1.0	1 :1.2
Age, mean (range)^b^	38 (2–96)	36 (3–75)	18 (3–45)	45 (27–75)	14 (2–44)
Age of onset, mean (range)^b^	16 (0–75)	14 (0–64)	7 (0–37)	24 (0–64)	6 (0–44)
Smoking status, % (never smoker; former smoker; current smoker)^c^	54; 28; 18	51; 27; 22	82; 6; 12	36; 37; 27	82; 7; 11
Clinical descriptive data					
FEV_1_, L (SD)^d^	2.93 (0.82)	2.99 (0.76)	2.93 (0.80)	3.01 (0.74)	2.93 (0.88)
FVC, L (SD)^e^	3.73 (1.02)	3.82 (0.94)	3.71 (1.04)	3.87 (0.88)	3.52 (1.10)
FEV_1_/FVC, % (SD)^f^	94.0 (9.0)	72.5 (22.4)	70.8 (29.6)	72.6 (21.2)	77.7 (21.1)
Serum IgE (SD)^g^	471 (1564)	432 (1406)	806 (2309)	251 (501)	276 (404)
Asthma, *n* (%)^h^	592 (49)	264 (59)	149 (100)	116 (36)	52 (47)
Allergy, *n* (%)^i^	677 (57)	287 (64)	121 (82)	170 (57)	73 (68)
With asthma, *n* (%)	433 (36)	206 (46)	121 (82)	90 (30)	37 (35)
Eosinophils^j^					
Count in 1e-9/L (SD)	0.24 (0.22)	0.25 (0.23)	0.32 (0.34)	0.21 (0.15)	0.26 (0.26)
Percentage (SD)	3.6 (2.8)	3.7 (2.8)	4.4 (3.3)	3.3 (2.4)	4.0 (3.4)

^a^Probands are the first family member recruited in the cohort

^b^Mean and median age calculated for 1212 subjects, 447 trios members, 149 probands, 298 parents and 110 siblings

^c^Smoking status was available for 1194 subjects, 444 trios members, 148 probands, 296 parents and 107 siblings. Ex-smokers are defined as subject who stopped smoking since over 1 year

^d^The mean forced expiratory volume in 1 s (FEV_1_) is measured in L in 925 subjects, 429 trios members, 141 probands, 287 parents and 94 siblings

^e^The mean forced vital capacity (FVC) is measured in L in 908 subjects, 414 trios members, 134 probands, 279 parents and 92 siblings

^f^The mean FEV_1_ (L)/FVC (L) ratio is calculated in % for 907 subjects, 414 trios members, 134 probands, 279 parents and 93 siblings

^g^The geometric mean of immunoglobulin (Ig) E serum concentration is calculated for 996 subjects, 408 trios members, 142 probands, 292 parents and 99 siblings

^h^Present or past documented clinical history of asthma. Asthma phenotype is available for 1207 subjects, 447 trio members, 149 probands, 298 parents and 110 siblings

^i^Allergy is defined as one positive skin prick testing (wheal diameter ≥ 3 mm at 10 min). The allergy phenotype is available for 1193 subjects, 445 trio members, 147 probands, 296 parents and 106 siblings

^j^Cell type profiles are available for 967 subjects, 418 trios members, 137 probands, 283 parents and 98 siblings

### Capture and sequencing

Samples from the 149 trios from the SLSJ asthma familial cohort and 93 non-related individuals from each of the four European populations were all sequenced using a custom capture panel developed by our group [22], followed by next-generation sequencing. This custom capture panel covers around 3% of the genome, including coding and non-coding immune regulatory regions [22] (see Morin et al. [22] for details about panel description, capture and sequencing) because most of the variants associated with complex traits are located in the non-coding region of the genome [23]. This approach allowed us to assess the impacts of rare and low-frequency variants in a cost-effective manner. Moreover, the use of next-generation sequencing was better suited in this context compared with genotyping chips or linkage analysis due to the small effect size and low-frequency of the variants we explored, as well as the low heritability of the traits [24]. To remove any potentially related individuals in the paired 465 subjects (93 subjects from each of the five populations), we performed an identity-by-descent estimation using the method of moments [25] and a principal component analysis using the SNPRelate R package [26] (Supplementary Figure 1A). We also used heterozygous/homozygous proportion to identify and remove outliers and their paired samples (Supplementary Figure 1B, C and D). A total of 380 samples (76 samples per population) were analyzed for variants characterization at the end. Four hundred forty samples from the SLSJ trios were kept for the second part of the study.

### Variant calling and filtering

We aligned reads to the Genome Reference Consortium Human genome build 37 (GRCh37) using bwa 0.7.6a and we called variants using HaplotypeCaller v3.2 (GATK). We performed merge calling for all samples in the five populations and the 149 trios independently. Variants included in both sample sets met these criteria: (1) included within the targeted regions, (2) biallelic sites, (3) dp > 10× and gq > 35 in at least 90% of the samples. We removed the human leukocyte antigen region as it will be analyzed independently. We compared the sequenced data with genotyped data obtained for the 149 trios (447 individuals; see *Genotyping* section) as a quality control using heterozygous and biallelic variants that were both in captured regions and on the genotyping chip. Seven samples were removed due to a concordance of <95%. We also used the comparison between sequenced and genotyped data to set our filtering cut-off to read depth (dp) > 10 × , genotyping quality (gq) > 35; an accuracy and a sensitivity of > 95% were observed. We assessed Mendelian errors using VCFtools [27] and a parent/proband pair was excluded due to high error rate. Mendelian errors in the remaining samples were replaced by missing values. Sequences were inferred and imputed from the sequenced trios samples for the rest of the cohort (see *Inference and imputation* sections). Functional annotation was performed using SNPeff [28] and selectively constrained variants were identified with the Genomic Evolutionary Rate Profiling (GERP++ score) [29].

### Genotyping

DNA extraction, genotyping (Illumina 610K Quad Array) and variant filtering of the 1214 individuals from the SLSJ asthma familial cohort have been described previously [1, 30]. These samples include the 149 trios sequenced. Genotyping data were used for quality cut-offs assessment, inference and imputation of the sequence for the entire cohort.

### Inference and imputation

Genotype phasing was performed using SHAPEIT v2 and duoHMM [31–33] to consider familial structures. Pre-phasing was done using the trios (440 samples) using merged sequencing and genotyping data, as well as on the whole cohort using only genotyping data (1214 samples). We inferred the sequence in the non-sequenced siblings who were part of the same families as the trios. Chromosomes were separated by breakpoints that were identified using duoHMM and NUCFAMTOOLS [34]. We were able to reassemble the sequence in the siblings using informative markers (heterozygous in one parent and homozygous in the other) and we inferred on average 98.72% (96.89–99.33%) of the sequence at an accuracy of 99.67% (99.39–99.86%). We used the parental haplotypes (294 samples) as the reference panel to reduce the number of duplicated haplotypes and we imputed the sequence using IMPUTE2 [35] in the whole cohort. Missing genotypes from the inference were added using the imputed data. A total of 112,154 variants were imputed with a mean accuracy of 99.70% (98.55–99.98%) and retained variants with imputation quality of > 0.8 for a total of 112,083 variants. The imputation accuracy was measured by comparing imputed probands to their sequenced data.

### DNA methylation

We measured DNA methylation levels on a subset of individuals from the SLSJ asthma cohort using whole blood (167 samples) and isolated eosinophils from blood (24 samples). Eosinophil cells isolation was performed as described in Ferland et al. [36] and DNA extraction and sodium bisulfite conversion were described in Liang et al. [37]. Methylation levels were assessed using the Infinium HumanMethylation450 BeadChip array (Illumina, San Diego, CA, USA). Normalization steps were described in Morin et al. [38].

### Statistical analyses

To assess variants distribution differences across the five populations, the variant proportions or ratios were compared using a chi-square test combined with Cramer’s V ( > 0.15 for 4 degrees of freedom) and an analysis of variance (ANOVA) followed by Tukey's test to assess the differences of per sample distribution. *p*-Values reported for the post hoc tests are adjusted for false discovery rate (chi-square) or with Bonferroni correction (ANOVA).

We performed single-variant analyses on low-frequency variants (0.01 < minor allele frequency (MAF) < 0.05) and which were not common in the UK10K [39] or 1000 Genomes Project (1KGP) [40] for a total of 15,294 variants (significance threshold set to *p* < 3.3e–6). For the gene-based test, we combined rare and low-frequency variants per gene, including its surrounding 20 kb (including only immune DNase I Hypersensitivity Sites (DHSs)) and only included gene regions that had at least two variants for a total of 14,646 (significance threshold set to *p* < 3.4e–6 or false discovery rate 5%).

We explored variants associated with the five investigated phenotypes using EPACTS software (Efficient and Parallelizable Association Container Toolbox: http://genome.sph.umich.edu/wiki/EPACTS). We performed a mixed model association called EMMAX (Efficient Mixed Model Association eXpedited) that accounts for sample structure (population structure and relatedness (kinship coefficient)). EMMAX supports single-variant association tests and different burden tests (combined multivariate and collapsing (CMC) [41] and Sequence kernel association test (SKAT) [42] methods). The two burden tests were used because of their different assumptions: CMC performs a multivariate test on the variant counts in each gene region [41], whereas SKAT combines the score of each variant in the gene regions assuming their independence, thus allowing them to go in different directions of effect [42]. Sex and age were used as covariates, as well as height for lung function assessment.

To determine if DNA methylation levels at genes associated with asthma-related traits were associated with serum levels, FEV_1_/FVC and asthma using whole blood (167 samples) and blood isolated eosinophils (24 samples), we applied a robust linear regression model including age and sex as covariates, as well as cell type composition for whole blood. To assess if results were obtained by chance, we performed 1000 permutations and determined if we get the same number of CpGs with *p* < 0.05.

Data are available through EGA (https://ega-archive.org/) for the SLSJ cohort (DAC: EGAC00001000901, Study: EGAS00001003103) and for the other cohorts (DAC: EGAC00001000409, Study: EGAS00001001564).

## Results

### Founder populations are enriched with population-specific low-frequency variants

**Fig. 1:**
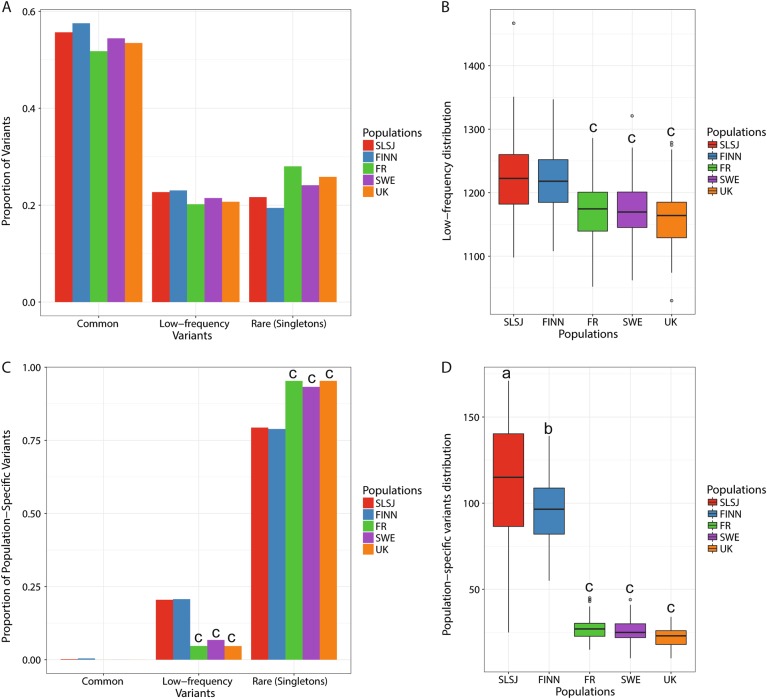
Distribution of variants across founder populations compared with three other European populations. **a** Proportion of common (MAF > 0.05), low-frequency (0.01 < MAF < 0.05) and rare (singletons) (MAF < 0.01) variants in each population; **b** Low-frequency variants distribution per sample, ANOVA *p* < 2e–16 and Tukey *p* < 1e–6; **c** Proportion of population-specific variants, common (MAF > 0.05), low-frequency (0.01 < MAF < 0.05) and rare (singletons; MAF < 0.01), chi-square *p* < 2e–16 and Cramer’s V = 0.24 for both low-frequency and rare variants and; **d** Population-specific variants distribution (not including singletons), ANOVA *p* < 2e–16 and Tukey *p* < 1e–3. Population-specific variants are defined as variants observed in only one of the five populations but are not necessarily singletons. To assess significance, chi-square test (*p* < 0.05) and Cramer’s V ( > 0.15) were performed for **a** and **c** and ANOVA followed by Tukey were performed for **b** and **d**. a = significantly different from FINN, b = significantly different from SLSJ, c = significantly different from FINN and SLSJ. In **c**, SWE is also significantly different from UK and FR. SLSJ Saguenay–Lac-Saint-Jean, FINN Finland, FR France, SWE Sweden, UK United Kingdom

For the first objective of characterizing variants distribution, after removal of related individuals and outliers 76 samples from the SLSJ cohort were paired with 76 samples from each of the four other European populations (a total of 380 individuals). All five populations had a mean coverage of 28 × (18–52 × ) (Table 2). A total of 192,227 variants (178,613 single-nucleotide variants (SNVs) and 13,614 indels) were called according to the aforementioned criteria and summarized in Table 2. Given the small number of samples being analyzed in this study, the rare variant spectrum only includes singletons, which are rare variants seen only one time. We observed a smaller number of variants in both founder populations (total number of variants: 111,669 (SLSJ), 108,485 (FINN), 120,473 (FR), 114,468 (SWE) and 116,937 (UK); Table 2) and was reflected in the mean number of singletons per sample for each population (Supplementary Table 1 and Supplementary Figure 2). A smaller proportion of singletons was observed in the SLSJ (21.6%) and FINN (19.4%) populations compared with other populations (28%, 24% and 25% for FR, SWE and UK respectively; Fig. 1a). Similar proportions of low-frequency variants were observed across each population (Fig. 1a). We observed a higher number of low-frequency variants in the founder populations when the mean number of variants per sample were compared with the non-founder populations (ANOVA *p* < 2e–16; Fig. 1b). For population-specific variants, which were only observed in one population, higher proportions of low-frequency variants were found in the SLSJ and FINN populations (chi-square *p* < 2e–16, Fig. 1c) and higher mean numbers per sample per population than the non-founder populations (ANOVA *p* < 2e–16, Fig. 1d) compared with the smaller proportion observed previously in public databases (chi-square *p* < 2e–16; Supplementary Figure 3B). For rare population-specific variants, we observed smaller proportion in SLSJ and FINN than the non-founder populations (chi-square *p* < 2e–16, Fig. 1c). For functional variants in the SLSJ and Finnish founder populations, although not significant, we observed a larger proportion of nonsynonymous, loss-of-function (LoF) and GERP++ > 4 low-frequency variants (Supplementary Figures 4 and 5 and Supplementary Tables 2 and 3), as well as a higher nonsynonymous/synonymous ratio trend and GERP++ > 4/GERP++ < 2 in the low-frequency variants of the founder populations compared with the others (Supplementary Figure 6A and 7A). The trend was significant when looking at the mean ratios of variants per sample for each population (ANOVA *p* = 3.72e–10 and *p* = 1.68e–9; Supplementary Figure 6C and 7C). We also looked at the enrichment of deleterious variants in the founder populations as compared with the non-founder populations (Supplementary Figure 8). SLSJ has significant excess of nonsynonymous low-frequency variants as compared with FR and UK (chi-square *p* = 8.3e–3 and *p* = 0.019; respectively, Supplementary Figure 8A) and excess low-frequency GERP++ > 4 variants than FR and UK (chi-square *p* = 2.9e–3 and *p* = 0.048; respectively, Supplementary Figure 8C). Similar patterns were observed for FINN; FINN has excess low-frequency nonsynonymous variants than FR (chi-square *p* = 0.048, Supplementary Figure 8B) and excess low-frequency GERP++ > 4 variants than FR and UK (chi-square *p* = 5.6e–4 and *p* = 0.014, respectively; Supplementary Figure 8D). Similar patterns were observed for population-specific variants (Supplementary Figure 9). No difference was observed for the average GERP++ score per sample distribution among populations (Supplementary Figure 10). However, greater average GERP++ scores in the low-frequency variants were observed in SLSJ and FINN as compared with FR and the UK (ANOVA *p* = 1.8e–7, Supplementary Figure 11A). Overall, we observed a higher proportion of population-specific variants reaching higher frequencies in the founder populations. We also observed a tendency of enrichment for more deleterious variants in the founder population, especially in the low-frequency spectrum.

**Table 2:** Overall description of variants included in the analyses

	All	SLSJ	FINN	FR	SWE	UK
Mean coverage	28.83	28.88	28.86	28.79	28.81	28.82
Ts/Tv	2.24	2.25	2.25	2.24	2.24	2.25
Total SNVs	178,613	103,889	100,696	112,047	106,375	108,511
Total indels	13,614	7780	7789	8426	8093	8426
Total singleton^a^	420	371	377	425	354	372

*SLSJ* Saguenay–Lac-Saint-Jean, *FINN* Finland, *FR* France, *SWE* Sweden, *UK* United Kingdom, *Ts/Tv* transition to transversion ratio, *Indels* insertions and deletions

^a^Singletons in the column entitled “All” corresponds to singletons across populations. The other columns correspond to singletons within each population. Each population comprise 76 samples for a total of 380 samples

### Low-frequency variant located in *CXCR6*/*FYCO1* is associated with eosinophil percentage in the SLSJ founder population

**Fig. 2:**
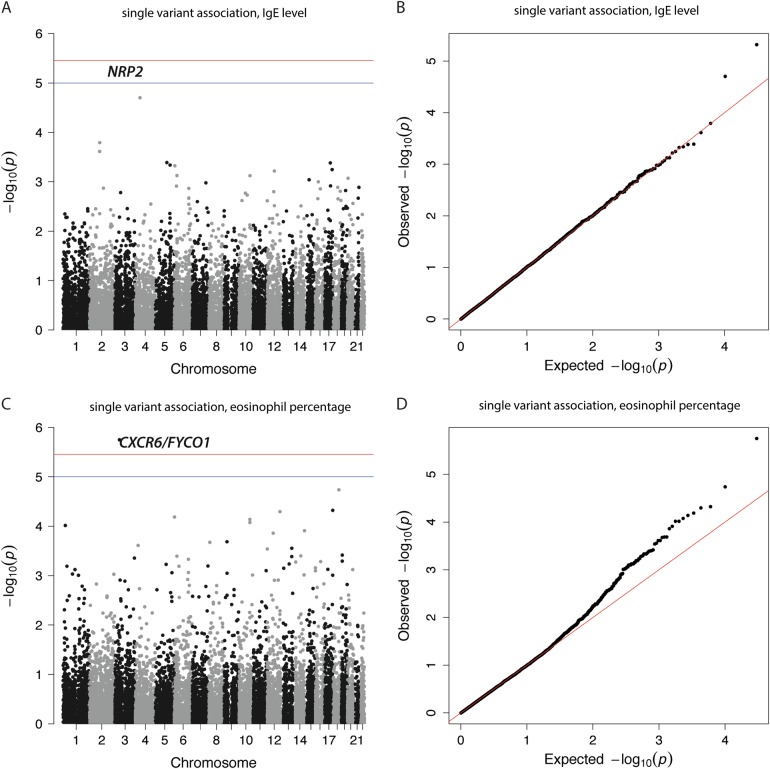
Manhattan and qqplot plot for low-frequency single-variant association test for **a** serum IgE levels (lambda = 1.01, with associated qqplot (**b**)); and **c** eosinophil percentage (lambda = 1.08, with associated qqplot (**d**)). Significance cut-off are shown in blue (*p* < 1e–5) and red (Bonferroni, *p* < 3.3e–6)

We assessed the individual effect of the coding and non-coding low-frequency (0.01 < MAF < 0.05) that were not common in the UK10K [39] or 1KGP [40]. A total of 15,294 SNVs (Bonferroni threshold: *p* < 3.26e–6) were analyzed for association with five asthma-related traits in the SLSJ cohort. We observed a significant association (*p* < 3.26e–6) with eosinophil percentage for SNV rs1386931:C > T (*p* = 1.77e–6, effect size = 1.534), located in the 3ʹ-UTR of *CXCR6* and in the intron of the *FYCO1* genes (Table 3 and Fig. 2c). We also observed another SNV reaching suggestive significance (*p* < 1e–5) with serum IgE levels and it is located in the intron of the *NRP2* gene (rs849558:T > C, *p* = 4.79e–6, effect size = −1.243; Table 3 and Fig. 2a). No variants were identified to be associated with lung function. Both SNVs were also found in 1KGP and UK10K. The SNV associated with eosinophil percentage (rs1386931:C > T) has a higher MAF in SLSJ (0.043) compared with the one observed in 1KGP (0.021) and UK10K (0.019). The other variant (rs849558:T > C) had slightly higher frequency in SLSJ (0.019) compared with UK10K (0.011).

**Table 3:** Results of single low-frequency SNV association study with asthma-related traits (*p* < 1e–5)

Trait	rsID	Gene	Alleles	MAF	*p*-Value^a^	Effect (SE)
Serum IgE levels	chr2:g.206562250 T > C; rs849558	*NRP2*; intron	T/C	0.019	4.79e–6	–1.243 (0.270)
Eosinophils percentage	chr3:g.45989502 C > T; rs1386931	*CXCR6*; 3ʹ-UTR and *FYCO1*; intron	C/T	0.043	**1.77e–6**	1.534 (0.319)

*MAF* minor allele frequency, *SE* standard error, *IgE* immunoglobulin E, *UTR* untranslated region, *NRP2* neuropilin 2, *CXCR6* C-X-C motif chemokine receptor 6

^a^ *p*-Value in bold is those reaching the significance threshold of 3.3e–6

### *NRP2* and *MRPL44* are associated with serum IgE levels and eosinophil percentage in the SLSJ cohort, respectively

**Fig. 3:**
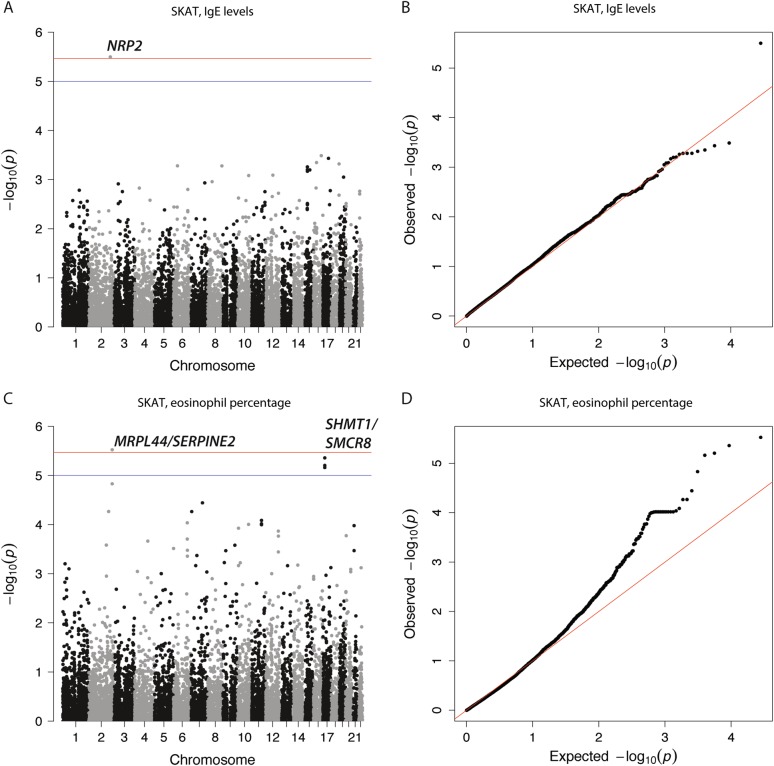
Manhattan plot and qqplot for SKAT test with **a** serum IgE levels (lambda = 1.05, with associated qqplot (**b**)) and **c** eosinophil percentage (lambda = 1.13, with associated qqplot (**d**)). SKAT Sequence Kernel Association Test. Significance cut-off are shown in blue (*p* < 1e–5) and red (Bonferroni, *p* < 3.4e–6)

For the gene-based test, we combined rare and low-frequency variants per gene, including its surrounding 20 kb (including only immune DHSs) and only included gene regions that had at least two variants for a total of 14,646 genes (*p* < 3.4e–6 or FDR 5%). Two genes were significantly associated with serum IgE levels (*NRP2, p* = 3.16e–6; Fig. 3a and Table 4) and eosinophil percentage (*MRPL44; p* = 2.97e–6; Fig. 3c and Table 4). One SNV lead each association: a rare one for *MRPL44* (rs76568361:T > G) and a low-frequency one for *NRP2* (rs849558:T > C; Table 4). The latter almost reached significance in the single-variant association test (*p* = 4.79e–6, Table 3). Moreover, we identified four marginally associated genes (*p* < 1e–5, Supplementary Table 4). *SHMT1* and *SMCR8* were marginally associated with eosinophil percentage (*p* = 2.97e–6 and *p* = 6.21e–6; Fig. 3c) with the rare nonsynonymous variant rs79875842:A > G being the lead SNV for both genes (Supplementary Table 4); *CCDC126* and *CLK2P* were marginally associated with FEV_1_/FVC (*p* = 4.62e–6; Supplementary Figure 12A) with rare nonsynonymous variants (rs73077128:G > A and rs146336907:C > T) for the pseudogene *CLK2P* (Supplementary Table 4). Variant rs146336907:C > T was not observed in 1KG or UK10K (Supplementary Table 4).

**Table 4:** Genes significantly associated with asthma and allergy-related traits (*p* < 3.4e–6)

Trait	Gene	*n* SNPs	*n* passing^a^	Fraction with rare	*p*-Value SKAT/CMC^b^	Lead SNVs^c^
Eosinophils percentage	*MRPL44*	8	4	0.026	**2.97e–6**/5.74e–5	chr2:g.224835223 T > G; rs76568361
Serum IgE levels	*NRP2*	4	2	0.001	**3.16e–6**/0.8237	chr2:g.206562250 T > C; rs849558

*SKAT* sequence kernel association test, *CMC* combined multivariate and collapsing test, *SNV* single-nucleotide variations, *IgE* immunoglobulin E, *NRP2* neuropilin 2, *MRPL44* mitochondrial ribosomal protein L44

^a^ Number of variants passing threshold (MAF < 0.05)

^b^ *p*-Values in bold are those reaching the significance threshold of 3.4e–6

^c^ Test were ran again removing one variant at a time, lead SNV correspond to the one for which the entire association rely on

### DNA methylation in associated genes

To support our associations observed from single variants and gene-based association test, we performed DNA methylation analyses of CpG located ± 20 kb from the associated genes (Supplementary Table 5) in whole blood (167 samples) or isolated eosinophils. The methylation levels of 49 CpG sites of *NRP2* were tested for association with serum IgE levels and nine CpGs with *p* < 0.05 (one with *p* < 2.8e–4) were detected, which is larger than expected (4.8 CpG) in isolated eosinophils (Supplementary Table 5). No associations were found between *CXCR6*/*FYCO1*, *MRPL44* and *SHMT1*/*SMCR8* methylation levels and asthma nor *CCDC26*/*CLK2P* with FEV_1_/FVC (Supplementary Table 5).

## Discussion

In our study, we characterized the variants distribution of rare and low-frequency variants in the SLSJ population, the enrichment of deleterious variants and their impacts on asthma and allergy-related traits. In terms of the variants distribution, overall observations in FINN were consistent with the published findings of another Finnish population genetic study [14]; higher proportions of population-specific rare and low-frequency variants and fewer rare variants as compared with non-founder populations. Unlike the published findings [14], no differences in proportions were detected in FINN when variants were stratified by function; it is likely due to small sample sizes. Similar to the findings of another French-Canadian population genetic study [13], a higher proportion of low-frequency variants was observed compared with non-founder populations. However, contrary to the same study [13], we observed a smaller proportion of rare population-specific variants in SLSJ and no difference in the proportion of low-frequency nonsynonymous variants as compared with non-founder populations; the latter likely due to a small sample size. However, a larger proportion of population-specific variants reached higher frequencies in the founder populations, reflecting the genetic drift associated with them. Our results are consistent with previous studies in terms of enrichments of deleterious variants in founder populations, especially in the low-frequency spectrum of variants [13, 14]. Various factors may contribute to the differences in the findings between our study and others. The strength of our assessment resides in comparing two founder and three non-founder populations with identical experimental procedures and samples paired based on mean coverage.

By investigating low-frequency variants in SLSJ, new genes have been identified for their associations with asthma or allergy-related traits. Using variant-based analyses, *FYCO1, CXCR6* and *NRP2* shed new light to mechanisms influencing allergy. Variant rs1386931:C > T is located in an intron of *FYCO1* and 3ʹ-UTR of the *CXCR6*. *FYCO1* encodes for a protein that plays a role in the transport of autophagic vesicles [43], and autophagy and its genes were associated with asthma [44–48]. *CXCR6* encodes for a chemokine receptor expressed on the surface of multiple immune cell types and was associated with asthma and Th2 inflammation in the lung [49, 50]. Variant rs849558:T > C is located in an intron of *NRP2*. It is a transmembrane receptor implicated in multiple processes, including antigen presentation, phagocytosis and cell–cell interaction [51]. This gene was also associated in the gene-based test; being led by the same associated SNV and was supported by DNA methylation association. Given that serum IgE levels and eosinophil percentage are associated with various allergic diseases, we tested variants rs849558:T > C and rs1386931:C > T for associations with additional disease phenotypes: asthma, atopy, allergic asthma, rhinitis and atopic dermatitis (Supplementary Table 6). *NRP2* variant rs849558:T > C was marginally associated with atopy and allergic asthma. *CXCR6/FYCO1* variant rs1386931:C > T was associated with atopic dermatitis. These findings point at plausible disease mechanisms of allergic diseases.

Using a gene-based approach, there are now suggestive roles of *MRPL44* and *SERPINE2* in mediating eosinophil percentage. *MRPL44* is implicated in protein synthesis in mitochondria and that mitochondria play an important role in eosinophil apoptosis and survival [52]. Moreover, the lead SNV for *MRPL44* is located in the promoter region of the *SERPINE2* gene, for which the association was suggestive (*p* = 1.47e–5). *SERPINE2* is a serine protease inhibitor and is a known susceptibility gene for chronic obstructive pulmonary disease [53], emphysema [54] and asthma [55]. Our findings suggest a novel role of SERPINE2 in eosinophils.

## Conclusion

In this study, we showed that founder populations are enriched with deleterious low-frequency variants and that they present population-specific variants with higher frequencies. By examining rare and low-frequency variants for their associations with asthma and allergy-related traits in a founder population, we identified new genes worthy of further study. One of the lead SNV in the gene-based test was specific to the SLSJ population highlighting the importance of using sequencing data in founder population to identify new genes associated with complex traits. Other SNV also presented marginally higher frequency compared with the European populations. We also demonstrate the importance of addressing the non-coding regions of the genome by using sequencing studies, as three of the variants identified were non-coding and located in immune regulatory regions. Overall, we showed the usefulness of using a well-described founder population and the importance of assessing non-coding regions to better decipher the genetic basis of complex traits.

## Electronic supplementary material

Supplementary information

Supplementary Figure 1. Samples selection from the five populations

Supplementary Figure 2. Mean number of singletons per sample for each population

Supplementary Figure 3. Proportion of all and population-specific variants previously observed in UK10K, 1000 Genomes Project, EXaC and dbSNP417

Supplementary Figure 4. Proportion of common (MAF>0.05), low-frequency (0.01<MAF<0.05) and rare (singletons; MAF<0.01) variants in each population

Supplementary Figure 5. Site frequency spectrum

Supplementary Figure 6. Non-synonymous to synonymous ratio

Supplementary Figure 7. Ratio of variants with GERP++>4 and GERP++<2

Supplementary Figure 8. Common, low-frequency and singleton variants enrichment for deleterious variants

Supplementary Figure 9. Population-specific low-frequency and singleton variants enrichment for deleterious variants

Supplementary Figure 10. Average GERP++ per sample distribution

Supplementary Figure 11. Average GERP++ per sample of low-frequency variants

Supplementary Figure 12. Manhattan plot and qqplot for CMC test with FEV_1_/FVC (Lambda= 1.04)
