# Supplementary material for: Exploring rare and low-frequency variants in the Saguenay–Lac-Saint-Jean population identified genes associated with asthma and allergy traits
Source: Eur J Hum Genet. 2018 Sep 11;27(1):90–101. doi: 10.1038/s41431-018-0266-4 (PMC6303288; doi:10.1038/s41431-018-0266-4)
Supplement: Supplementary file 1 — Supplementary information [file 41431_2018_266_MOESM1_ESM.docx]

**Exploring rare and low-frequency variants in the Saguenay–Lac-Saint-Jean population identified genes associated with asthma and allergy traits**

**Running Title: Rare & low-frequency variants in asthma & allergy**

Andréanne Morin^1,2^, Anne-Marie Madore^3^, Tony Kwan^1,2^, Maria Ban^4^, Jukka Partanen^5^, Lars Rönnblom^6^, Ann-Christine Syvänen^7^, Stephen Sawcer^4^, Hendrik Stunnenberg^8^, Mark Lathrop^1,2^, Tomi Pastinen^1,2,9,^, Catherine Laprise^3,10^*

1. Department of Human Genetics, McGill University, Montréal, Quebec, Canada.
2. McGill University and Genome Québec Innovation Centre, Montréal, Quebec, Canada.
3. Département des sciences fondamentales, Université du Québec à Chicoutimi, Saguenay, Quebec, Canada.
4. Department of Clinical Neurosciences, University of Cambridge, Cambridge, UK.
5. Research & Development, Finnish Red Cross Blood Service, Helsinki, Finland.
6. Department of Medical Sciences, Section of Rheumatology, Uppsala University, Uppsala, Sweden.
7. Department of Medical Sciences, Molecular Medicine and Science for Life Laboratory, Uppsala University, Uppsala, Sweden.
8. Department of Molecular Biology, Faculty of Science, Radboud University, Nijmegen, the Netherlands.
9. Center for Pediatric Genomic Medicine, Kansas City, Missouri, USA
10. Centre intégré universitaire de santé et de services sociaux du Saguenay, Saguenay, Quebec, Canada

*Corresponding author

## Supplementary information

Supplementary Table 1. Summary of variants in the five populations

|  |  | **All** | **SLSJ** | **FINN** | **FR** | **SWE** | **UK** |
| --- | --- | --- | --- | --- | --- | --- | --- |
| **Number of SNVs** | All | 178,613 | 103,889 | 100,696 | 112,047 | 106,375 | 108,511 |
|  | Common | 58,568 | 58,223 | 58,421 | 58,404 | 58,285 | 58,413 |
|  | Low-frequency | 21,949 | 23,362 | 22,899 | 22,362 | 22,527 | 22,229 |
|  | Rare | 98,096 | NA | NA | NA | NA | NA |
|  | Singletons | 64,834 | 22,304 | 19,376 | 31,281 | 25,563 | 27,869 |
| **Number of indels** | All | 13,614 | 7,780 | 7,789 | 8,426 | 8,093 | 8,259 |
|  | Common | 3,981 | 3,936 | 3,970 | 3,998 | 4,016 | 4,014 |
|  | Low-frequency | 2,019 | 1,966 | 2,087 | 1,976 | 2,038 | 1,947 |
|  | Rare | 7,614 | NA | NA | NA | NA | NA |
|  | Singletons | 4,852 | 1,878 | 1,704 | 2,480 | 2,039 | 2,298 |

SLSJ: Saguenay–Lac-Saint-Jean, FINN: Finland, FR: France, SWE: Sweden, UK: United Kingdom, SNVs: single nucleotide variations, indels: insertions and deletions.

Supplementary Table 2. Summary of functional variants in the five populations

|  |  | **SLSJ** | **FINN** | **FR** | **SWE** | **UK** |
| --- | --- | --- | --- | --- | --- | --- |
| **All** | Synonymous | 9,323 | 9,088 | 10,197 | 9,538 | 9,750 |
|  | Non-Synonymous | 12,094 | 11,707 | 13,249 | 12,511 | 12,673 |
|  | LoF | 459 | 443 | 486 | 456 | 470 |
| **Common** | Synonymous | 5,217 | 5,275 | 5,242 | 5,211 | 5,238 |
|  | Non-Synonymous | 5,508 | 5,638 | 5,646 | 5,576 | 5,609 |
|  | LoF | 160 | 164 | 166 | 160 | 160 |
| **Low-frequency** | Synonymous | 2,120 | 2,072 | 2,084 | 2,107 | 2,054 |
|  | Non-Synonymous | 3,089 | 2,993 | 2,758 | 2,904 | 2,762 |
|  | LoF | 129 | 119 | 95 | 116 | 105 |
| **Singletons** | Synonymous | 942 | 770 | 1,669 | 988 | 1,277 |
|  | Non-Synonymous | 1,937 | 1,578 | 2,990 | 2,083 | 2,374 |
|  | LoF | 110 | 100 | 148 | 104 | 136 |

LoF: Loss of function, SLSJ: Saguenay–Lac-Saint-Jean, FINN: Finland, FR: France, SWE: Sweden, UK: United Kingdom.

Supplementary Table 3. Summary of population-specific variants in the five populations

|  |  | **SLSJ** | **FINN** | **FR** | **SWE** | **UK** |
| --- | --- | --- | --- | --- | --- | --- |
|  | All | 14,909 | 12,037 | 20,513 | 13,839 | 16,677 |
|  | Common | 25 | 51 | 0 | 0 | 0 |
|  | Low-freq | 3,052 | 2,493 | 960 | 931 | 779 |
|  | Singletons | 11,832 | 9,493 | 19,553 | 12,908 | 15,898 |
| **All** | Synonymous | 1,189 | 962 | 1,756 | 1,057 | 1,329 |
|  | Non-Synonymous | 2,409 | 1,996 | 3,101 | 2,223 | 2,488 |
|  | LoF | 135 | 122 | 152 | 111 | 140 |
| **Common** | Synonymous | 0 | 3 | 0 | 0 | 0 |
|  | Non-Synonymous | 2 | 8 | 0 | 0 | 0 |
|  | LoF | 0 | 1 | 0 | 0 | 0 |
| **Low-frequency** | Synonymous | 247 | 189 | 87 | 69 | 52 |
|  | Non-Synonymous | 470 | 410 | 111 | 140 | 114 |
|  | LoF | 25 | 21 | 4 | 7 | 4 |
| **Singletons** | Synonymous | 942 | 770 | 1,669 | 988 | 1,277 |
|  | Non-Synonymous | 1,937 | 1,578 | 2,990 | 2,083 | 2,374 |
|  | LoF | 110 | 100 | 148 | 104 | 136 |

LoF: Loss of function, SLSJ: Saguenay–Lac-Saint-Jean, FINN: Finland, FR: France, SWE: Sweden, UK: United Kingdom.

**Supplementary Table 4.** Genes reaching p<1e-5 using CMC or SKAT in one of the five asthma and allergy related phenotypes

| **Trait** | **Gene** | **n SNPs** | **n passing^1^** | **Fraction with rare** | **P-value SKAT/CMC^2^** | **Lead SNVs^3^** | **P-value after removing lead SNV** | **MAF lead SNV (1KG and UK10K)** | **P-value single variant** |
| --- | --- | --- | --- | --- | --- | --- | --- | --- | --- |
| **Eosinophils**  **percentage** | *MRPL44* | 8 | 4 | 0.026 | **2.97e-6/**5.74e-5 | chr2:g.224835223 T>G; rs76568361 | 0.4425/0.4168 | 0.0067 (NA and 0.00026) | NA |
|  | *SHMT1* | 7 | 5 | 0.017 | 6.21e-6/3.13e-4 | chr17:g.18220268 A>G; rs79875842 | 0.7761/ 0.7371 | 0.0046 (0.0145 and 0.0148) | NA |
|  | *SMCR8* | 8 | 6 | 0.018 | 6.85e-6/3.66e-4 | chr17:g.18220268 A>G; rs79875842 | 0.8431/ 0.6837 | 0.0046 (0.0145 and 0.0148) | NA |
| **FEV_1_/FVC** | *CCDC126/ CLK2P* | 3 | 3 | 0.022 | 3.19e-5/4.62e-6 | Both SNV: chr7:g.23624887 G>A; rs73077128, chr7:g.23625481 C>T; rs146336907 | 8.93e-4 and 0.0018/ 8.65e-4 and 0.0018 | 0.0036 (0.0106 and 0.0082), 0.0062 (NA) | NA |
| **Serum IgE Levels** | *NRP2* | 11 | 8 | 0.224 | **3.16e-6**/0.8237 | chr2:g.206562250 T>C; rs849558 | 0.0371 (0.021 and 0.019) | 0.0191 | 4.80e-6 |

^1^ Number of variants passing threshold (MAF<0.05). ^2^ P values in bold are those reaching the significance threshold of 3.4e-6. ^3^ Test were ran again removing one variant at a time, lead SNV correspond to the one for which the entire association rely on. SKAT: sequence kernel association test, CMC: combined multivariate and collapsing test, SNV: single nucleotide variations, MAF: minor allele frequency, 1KG: 1000 genomes project, FEV_1_/FVC: Tiffeneau-Pinelli index, IgE: immunoglobulin E, MRPL44: mitochondrial ribosomal protein L44, SHMT1: serine hydroxymethyltransferase 1, SMCR8: Smith-Magenis syndrome chromosome region candidate 8, CCDC126: coiled-coil domain containing 126, CLK2P: CDC like kinase 2 pseudogene, NRP2: neuropilin 2.

**Supplementary Table 5.** DNA methylation in associated genes

|  |  |  | **Number of CpG with p<0.05 (p<2.8e-4)** | | | |
| --- | --- | --- | --- | --- | --- | --- |
| **Tissue** | **Gene** | **Number of CpGs^1^** | **Asthma^2^** | **IgE levels** | **FEV_1_/FVC** | **Randomly selected CpGs^3^** |
| Eosinophils | *CCDC126/CLK2P* | 34 |  |  | 2 (0) | 3.0 |
|  | *CXCR6/FYCO1* | 28 | 3 (0) |  |  | 2.5 |
|  | *MRPL44* | 25 | 2 (1) |  |  | 2.3 |
|  | *NRP2* | 49 |  | 9 (1) |  | 4.9 |
|  | *SHMT1/SMCR8* | 45 | 4 (0) |  |  | 4.1 |
| Whole-blood | *CCDC126/CLK2P* | 34 |  |  | 2 (0) | 3.6 |
|  | *CXCR6/FYCO1* | 28 | 1 (0) |  |  | 3.2 |
|  | *MRPL44* | 25 | 3 (0) |  |  | 2.9 |
|  | *NRP2* | 49 |  | 0 (0) |  | 2.1 |
|  | *SHMT1/SMCR8* | 45 | 5 (0) |  |  | 5.2 |

^1^ Number of CpGs located +/- 20 Kb from gene. ^2^ For the genes associated with eosinophil percentage, we tested the number of CpGs associated with asthma. ^3^ To test if results were obtained by chance, we resampled randomly 1,000 times the same number of CpG as observed in the vicinity of the gene and listed the average number of CpGs that reached p<0.05. FEV_1_/FVC: Tiffeneau-Pinelli index, IgE: immunoglobulin E, MRPL44: mitochondrial ribosomal protein L44, SHMT1: serine hydroxymethyltransferase 1, SMCR8: Smith-Magenis syndrome chromosome region candidate 8, CCDC126: coiled-coil domain containing 126, CLK2P: CDC like kinase 2 pseudogene, NRP2: neuropilin 2, CXCR6: C-X-C motif chemokine receptor 6, FYCO1: FYVE and coiled-coil domain 1.

**Supplementary Table 6.** Low-frequency variants reaching p<1e-5 in single variant association and their significance level (p-value) in other traits.

| **rsID** | **Gene** | **Asthma** | **Atopy** | **Allergic asthma** | **Rhinitis** | **Atopic dermatitis** | **Serum IgE Levels** | **Eosinophils**  **percentage** | **FEV_1_** | **FVC** | **FEV_1_/ FVC** |
| --- | --- | --- | --- | --- | --- | --- | --- | --- | --- | --- | --- |
| chr2:g.206562250 T>C; rs849558 | *NRP2*; intron | 0.7986 | **0.0040** | **0.0134** | 0.0946 | 0.0773 | **4.79e-6** | 0.0930 | 0.1959 | 0.2538 | 0.1996 |
| chr3:g.45989502 C>T; rs1386931 | *CXCR6*; 3’UTR and *FYCO1*; intron | 0.1658 | 0.2422 | 0.7746 | 0.8691 | **0.0353** | **0.0052** | **1.77e-6** | 0.3213 | 0.5572 | 0.5001 |

P values in bold are those with p<0.05. IgE: Immunoglobulin E, FEV_1_: Forced expiratory volume in one second, FVC: forced vital capacity, NRP2: neuropilin 2, CXCR6: C-X-C motif chemokine receptor 6, FYCO1: FYVE and coiled-coil domain 1.

Titles and legends to supplementary figures

Supplementary Figure 1. Samples selection from the five populations. A) principal component analysis (PCA); B) Identity-by-descent estimation using method of moments including all 380 samples; C) total number of variants compared to mean coverage; D) heterozygous to homozygous proportion compared to mean coverage. SLSJ: Saguenay–Lac-Saint-Jean, FINN: Finland, FR: France, SWE: Sweden, UK: United Kingdom

Supplementary Figure 2. Mean number of singletons per sample for each population. To assess significance, ANOVA followed by Tukey were performed. ANOVA p<2e-16 and Tukey p<1e-7. a= significantly different from FINN, b= significantly different from SLSJ, c= significantly different from FINN and SLSJ. SLSJ: Saguenay–Lac-Saint-Jean, FINN: Finland, FR: France, SWE: Sweden, UK: United Kingdom

**Supplementary Figure 3.** **Proportion of all and population-specific variants previously observed in UK10K, 1000 Genomes project, EXaC and dbSNP417.** A) All common, low-frequency (0.01<MAF<0.05) and rare variants (singletons; MAF<0.01); B) population-specific low-frequency (0.01<MAF<0.05; chi-square p<2.2e-16 and Cramer’s V =0.20) and rare variants (singletons; MAF<0.01). To assess significance, chi-square test (p<0.05) and Cramer’s V (>0.15) were performed. a= significantly different from FINN, b= significantly different from SLSJ, c= significantly different from FINN and SLSJ. In B, SWE is also significantly different from UK and FR. SLSJ: Saguenay–Lac-Saint-Jean, FINN: Finland, FR: France, SWE: Sweden, UK: United Kingdom.

**Supplementary Figure 4.** **Proportion of common (MAF>0.05), low-frequency (0.01<MAF<0.05) and rare (singletons; MAF<0.01) variants in each population**. A) Non-synonymous, B) synonymous, C) loss of function and D) GERP++>4 variants from each population. To assess significance, chi-square test (p<0.05) and Cramer’s V (>0.15) were performed. SLSJ: Saguenay–Lac-Saint-Jean, FINN: Finland, FR: France, SWE: Sweden, UK: United Kingdom.

**Supplementary Figure** **5**. **Site frequency spectrum**. A) All variants and B) non-synonymous variants using derived allele frequency from chimp ancestral allele. SLSJ: Saguenay–Lac-Saint-Jean, FINN: Finland, FR: France, SWE: Sweden, UK: United Kingdom.

Supplementary Figure 6. Non-synonymous to synonymous ratio. A) Total number per population, B) mean ratio per sample for each population, C) mean ratio of low-frequency variants per sample for each population, ANOVA p=3.7e-10 and Tukey p<0.01, and D) mean ratio of singletons per sample for each population, ANOVA p=4.5e-3 and Tukey p<0.01. To assess significance, chi-square test (p<0.05) and Cramer’s V (>0.15) were performed for A and ANOVA followed by Tukey (p<0.05) were performed for B, C and D. a= significantly different from FINN, b= significantly different from SLSJ, c= significantly different from FINN and SLSJ. SLSJ: Saguenay–Lac-Saint-Jean, FINN: Finland, FR: France, SWE: Sweden, UK: United Kingdom.

Supplementary Figure 7. Ratio of variants with GERP++>4 and GERP++<2. A) Total number per population, B) mean ratio per sample for each population, ANOVA p=8.7e-6 and Tukey p<0.05, C) mean ratio of low-frequency variants per sample for each population, ANOVA p=1.7e-9 and Tukey p<1e-4, and D) mean ratio of singletons per sample for each population, ANOVA p=3.7e-2 and Tukey p=3.1e-2. To assess significance, chi-square test (p<0.05) and Cramer’s V (>0.15) were performed for A and ANOVA followed by Tukey (p<0.05) were performed for B, C and D. a= significantly different from FINN, b= significantly different from SLSJ, c= significantly different from FINN and SLSJ. SLSJ: Saguenay–Lac-Saint-Jean, FINN: Finland, FR: France, SWE: Sweden, UK: United Kingdom.

Supplementary Figure 8. Common, low-frequency and singleton variants enrichment for deleterious variants. A) and C) Saguenay–Lac-Saint-Jean (SLSJ) and B) and D) Finland (FINN) compared to France (FR), Sweden (SWE) and United Kingdom (UK) for synonymous, non-synonymous and loss of function (LoF) variants (A and B) and GERP++>2 and >4 (C and D). To assess significance, chi-square test (p<0.05) and Cramer’s V (>0.15) were performed and * indicates significant results. The black line indicates equals proportions between populations (i.e. no enrichment).

Supplementary Figure 9. Population-specific low-frequency and singleton variants enrichment for deleterious variants. A) and C) Saguenay–Lac-Saint-Jean (SLSJ) and B) and D) Finland (FINN) compared to France (FR), Sweden (SWE) and United Kingdom (UK) for synonymous, non-synonymous and loss of function (LoF) variants (A and B) and GERP++>2 and >4 (C and D). To assess significance, chi-square test (p<0.05) and Cramer’s V (>0.15) were performed and * indicates significant results. The black line indicates equals proportions between populations (i.e. no enrichment).

Supplementary Figure 10. Average GERP++ per sample distribution. A) All, B) non-synonymous, C) loss of Function (LoF) and D) synonymous. To assess significance, ANOVA followed by Tukey (p<0.05) were performed. SLSJ: Saguenay–Lac-Saint-Jean, FINN: Finland, FR: France, SWE: Sweden, UK: United Kingdom.

**Supplementary Figure 11. Average GERP++ per sample of low-frequency variants.** A) All**,** ANOVA p=1.8e-7 and Tukey p<0.01, B) non-synonymous**,** ANOVA p=2.7e-4 and Tukey p<0.05, C) loss of Function, ANOVA p=4.5e-3 and Tukey p<0.01, and D) synonymous, ANOVA p=6.3e-3 and Tukey p<0.01. To assess significance, ANOVA followed by Tukey (p<0.05) were performed. a= significantly different from FINN, b= significantly different from SLSJ, c= significantly different from FINN and SLSJ. SLSJ: Saguenay–Lac-Saint-Jean, FINN: Finland, FR: France, SWE: Sweden, UK: United Kingdom.

**Supplementary Figure 12. Manhattan plot and qqplot for CMC test with FEV_1_/FVC (Lambda= 1.04).** CMC= Combined Multivariate and Collapsing test. Significance cut-off are shown in blue (p<1e-5) and red (Bonferroni, p<3.4e-6).
