# Supplementary figures and images for: Exploring rare and low-frequency variants in the Saguenay–Lac-Saint-Jean population identified genes associated with asthma and allergy traits
Source: Eur J Hum Genet. 2018 Sep 11;27(1):90–101. doi: 10.1038/s41431-018-0266-4 (PMC6303288; doi:10.1038/s41431-018-0266-4)

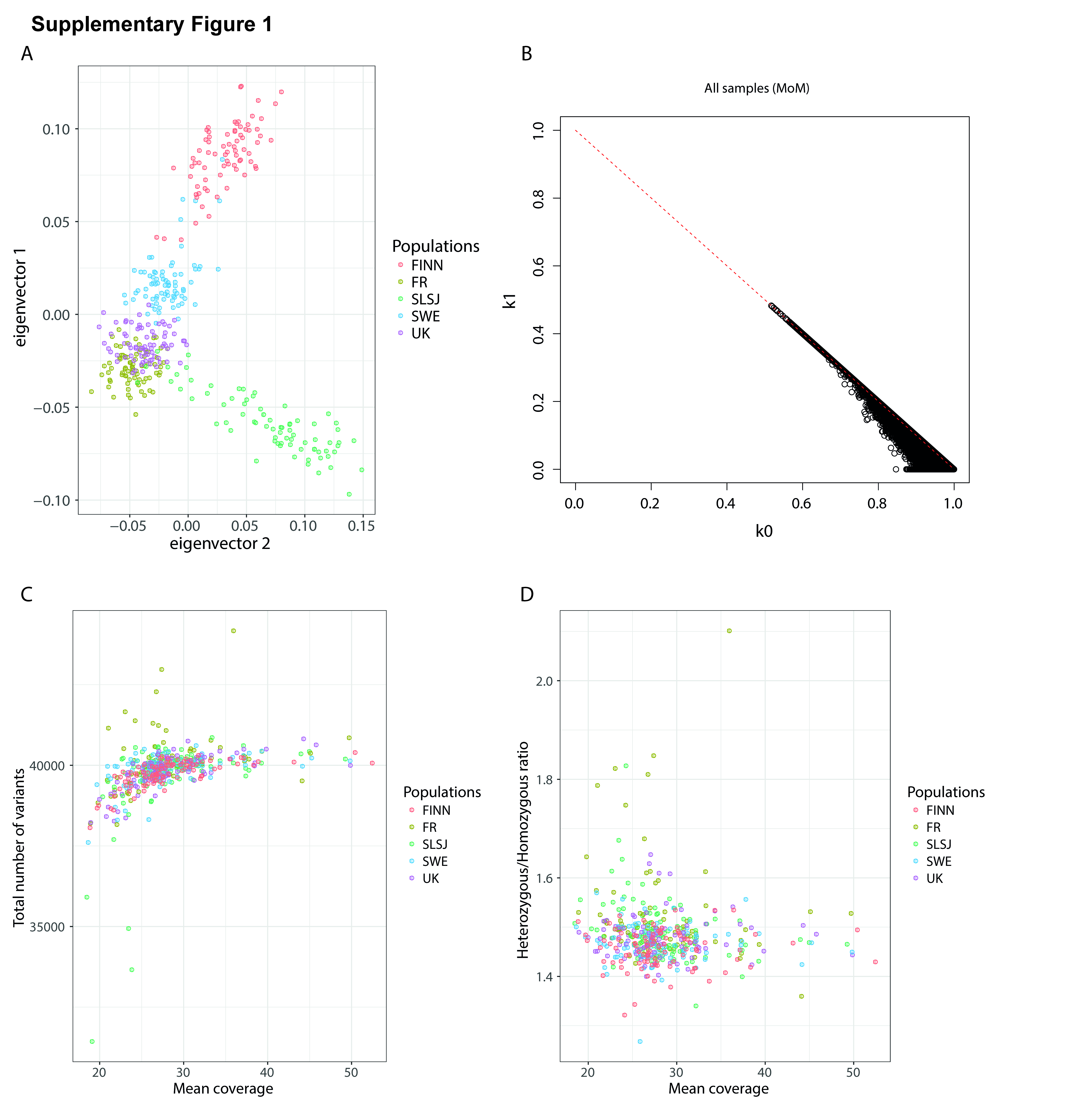

Supplement: Supplementary file 2 — Supplementary Figure 1. Samples selection from the five populations [file 41431_2018_266_MOESM2_ESM.tif]

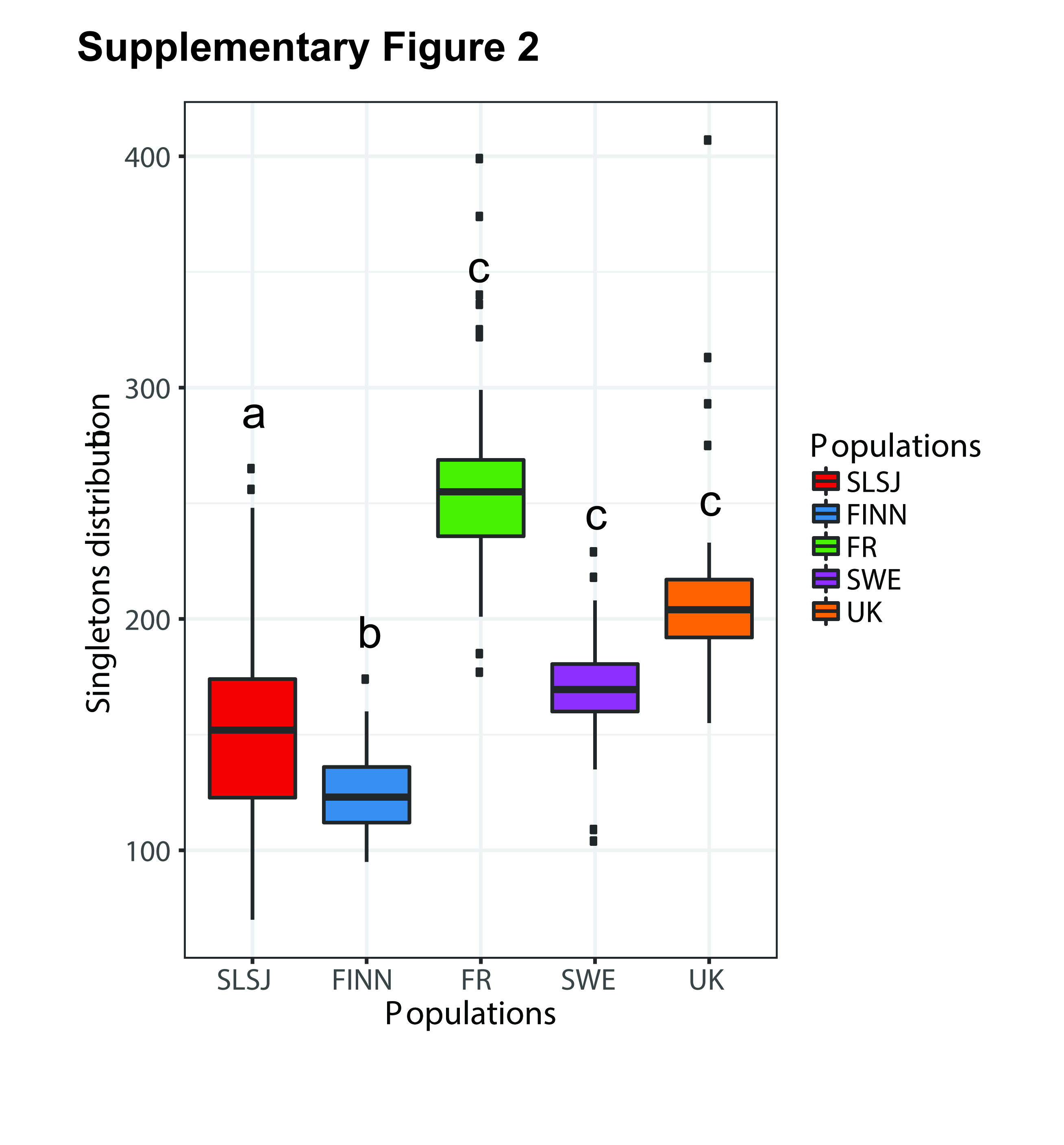

Supplement: Supplementary file 3 — Supplementary Figure 2. Mean number of singletons per sample for each population [file 41431_2018_266_MOESM3_ESM.tif]

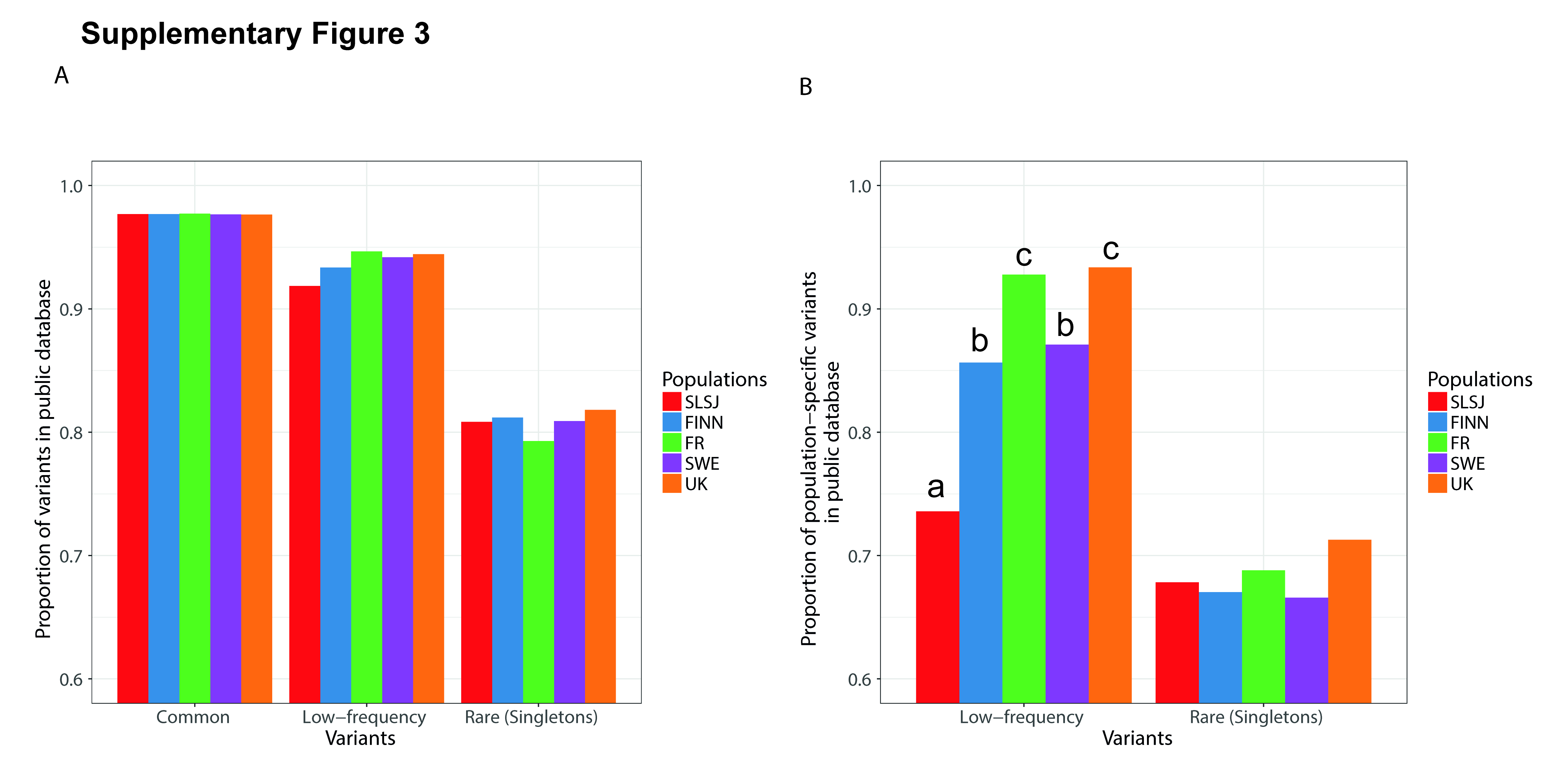

Supplement: Supplementary file 4 — Supplementary Figure 3. Proportion of all and population-specific variants previously observed in UK10K, 1000 Genomes Project, EXaC and dbSNP417 [file 41431_2018_266_MOESM4_ESM.tif]

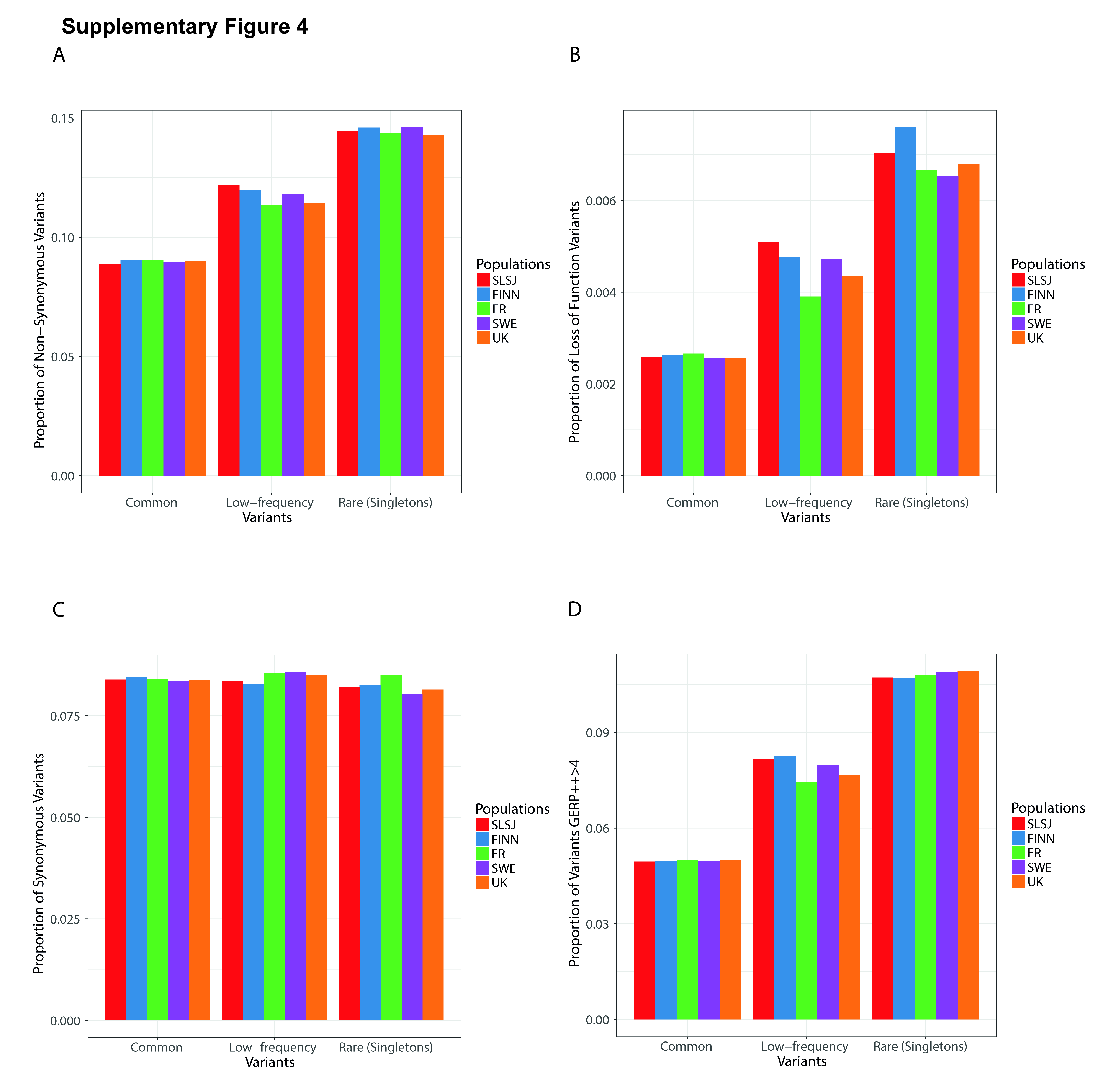

Supplement: Supplementary file 5 — Supplementary Figure 4. Proportion of common (MAF>0.05), low-frequency (0.01<MAF<0.05) and rare (singletons; MAF<0.01) variants in each population [file 41431_2018_266_MOESM5_ESM.tif]

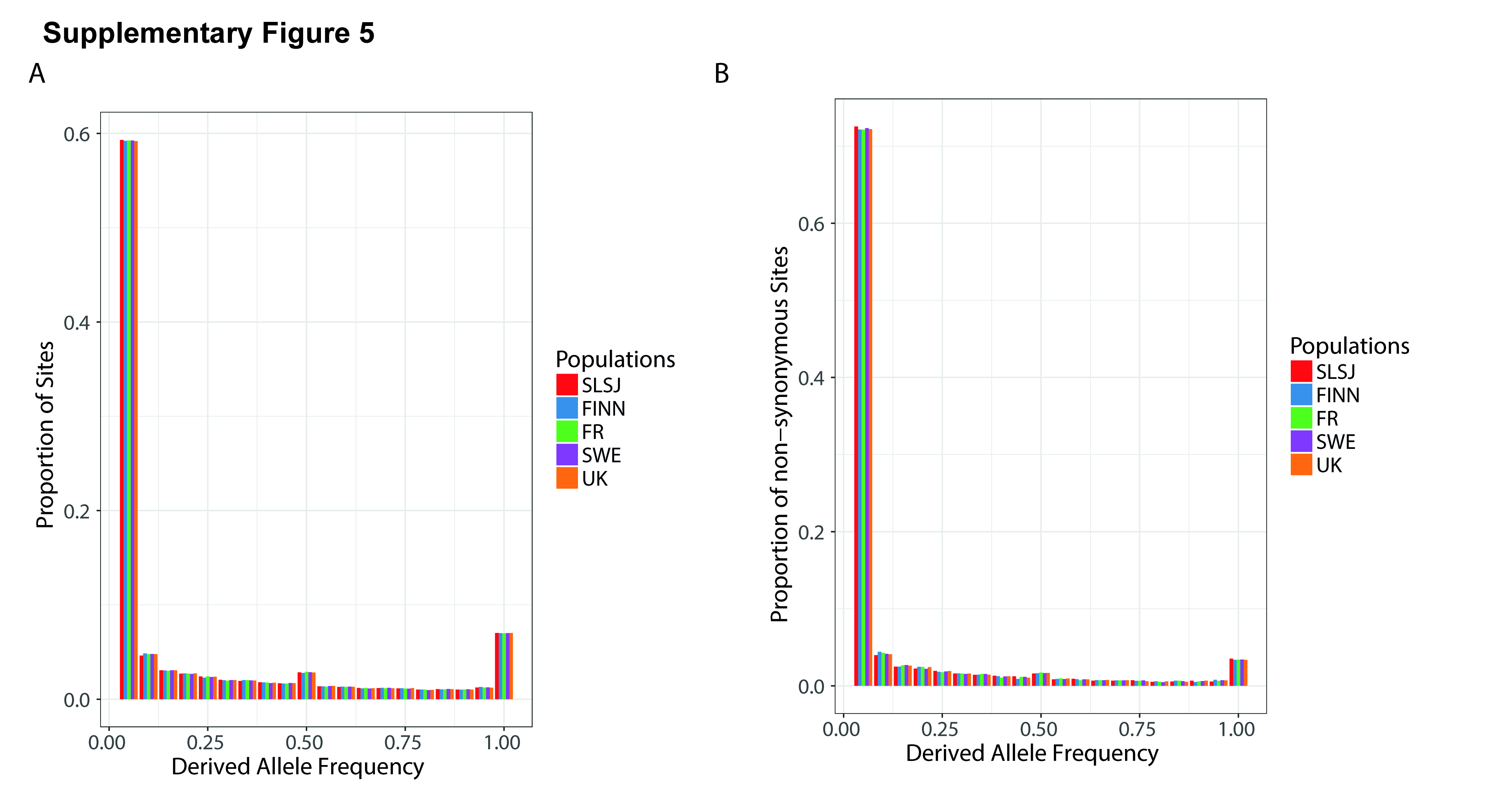

Supplement: Supplementary file 6 — Supplementary Figure 5. Site frequency spectrum [file 41431_2018_266_MOESM6_ESM.tif]

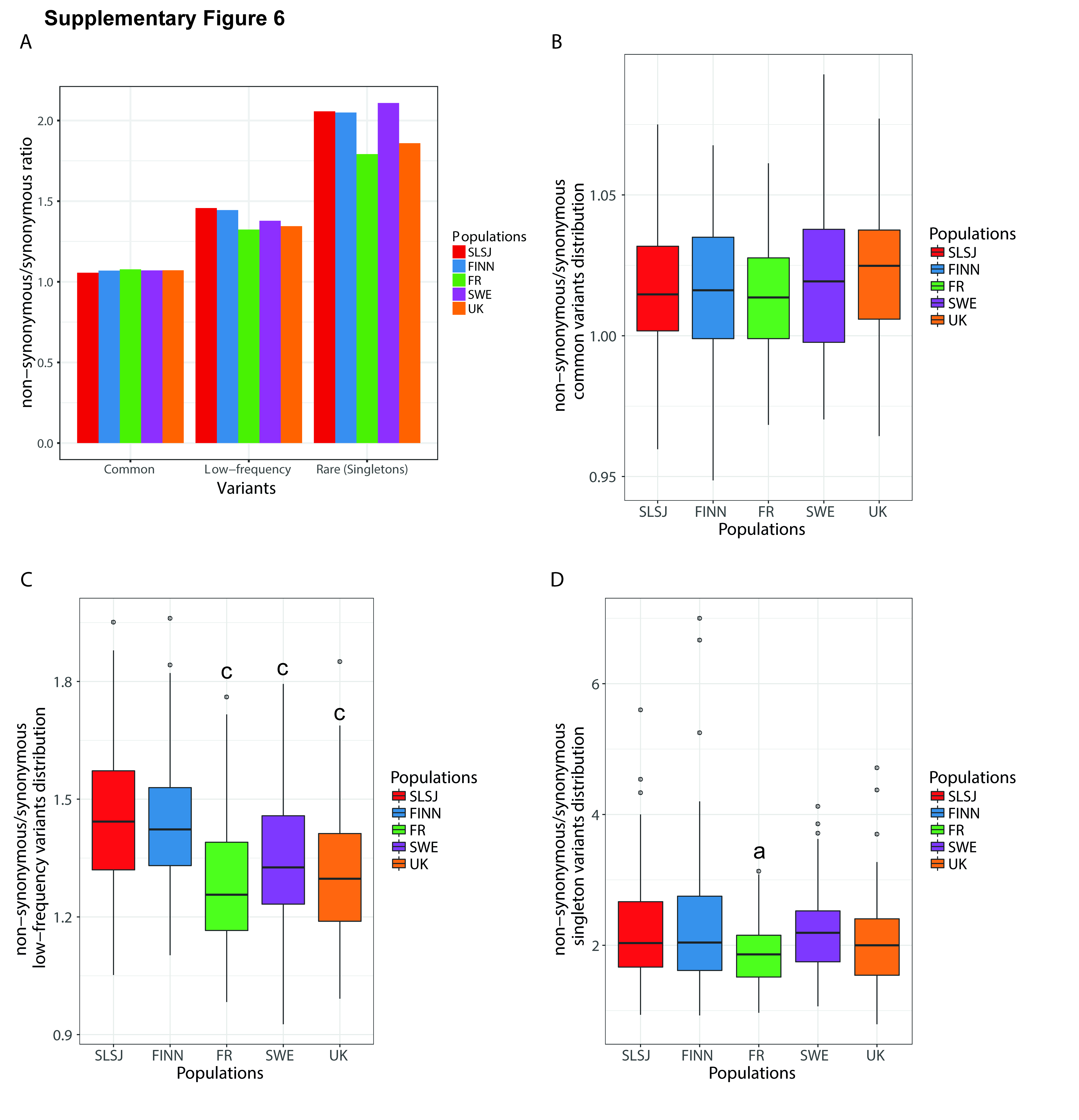

Supplement: Supplementary file 7 — Supplementary Figure 6. Non-synonymous to synonymous ratio [file 41431_2018_266_MOESM7_ESM.tif]

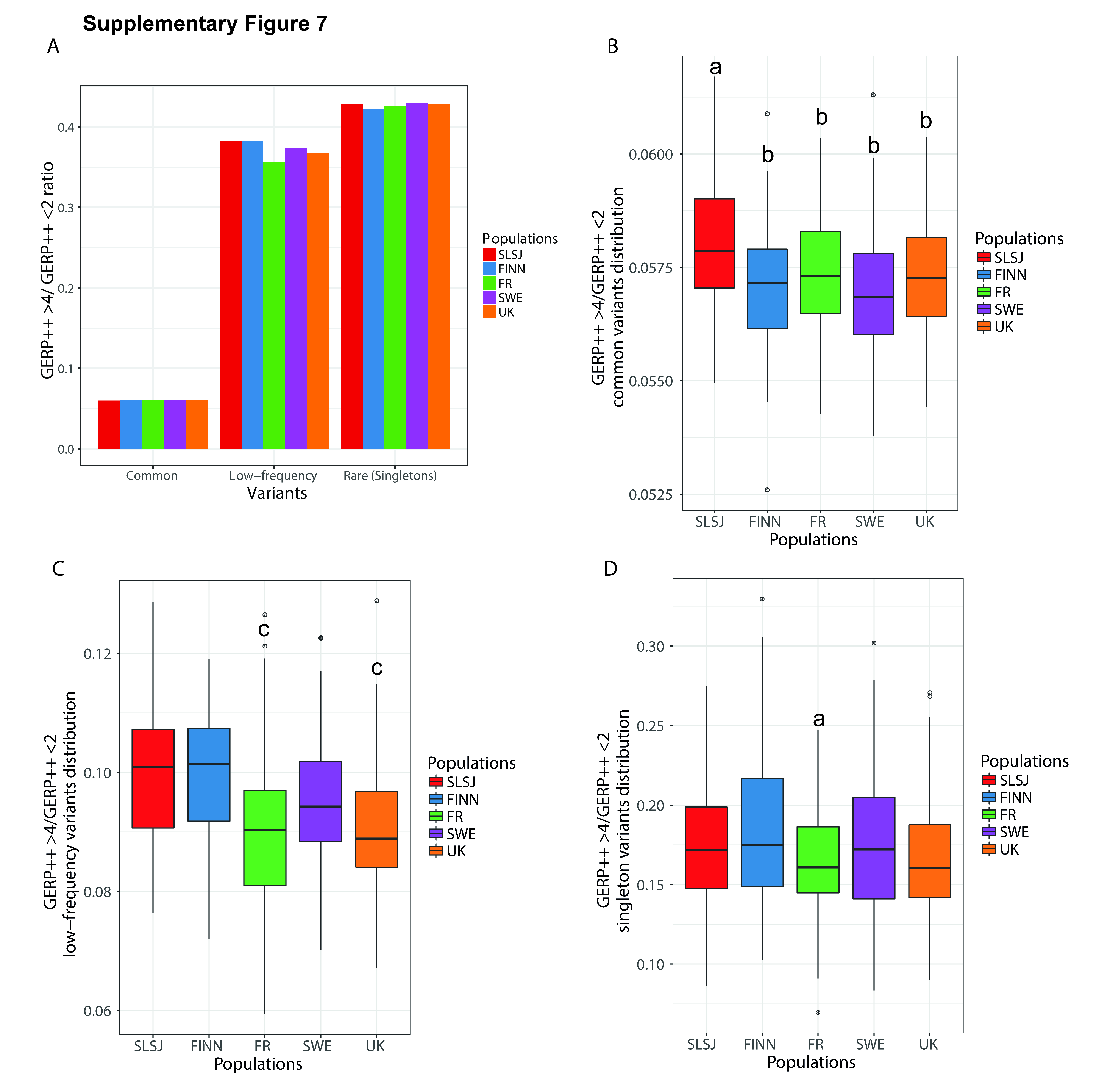

Supplement: Supplementary file 8 — Supplementary Figure 7. Ratio of variants with GERP++>4 and GERP++<2 [file 41431_2018_266_MOESM8_ESM.tif]

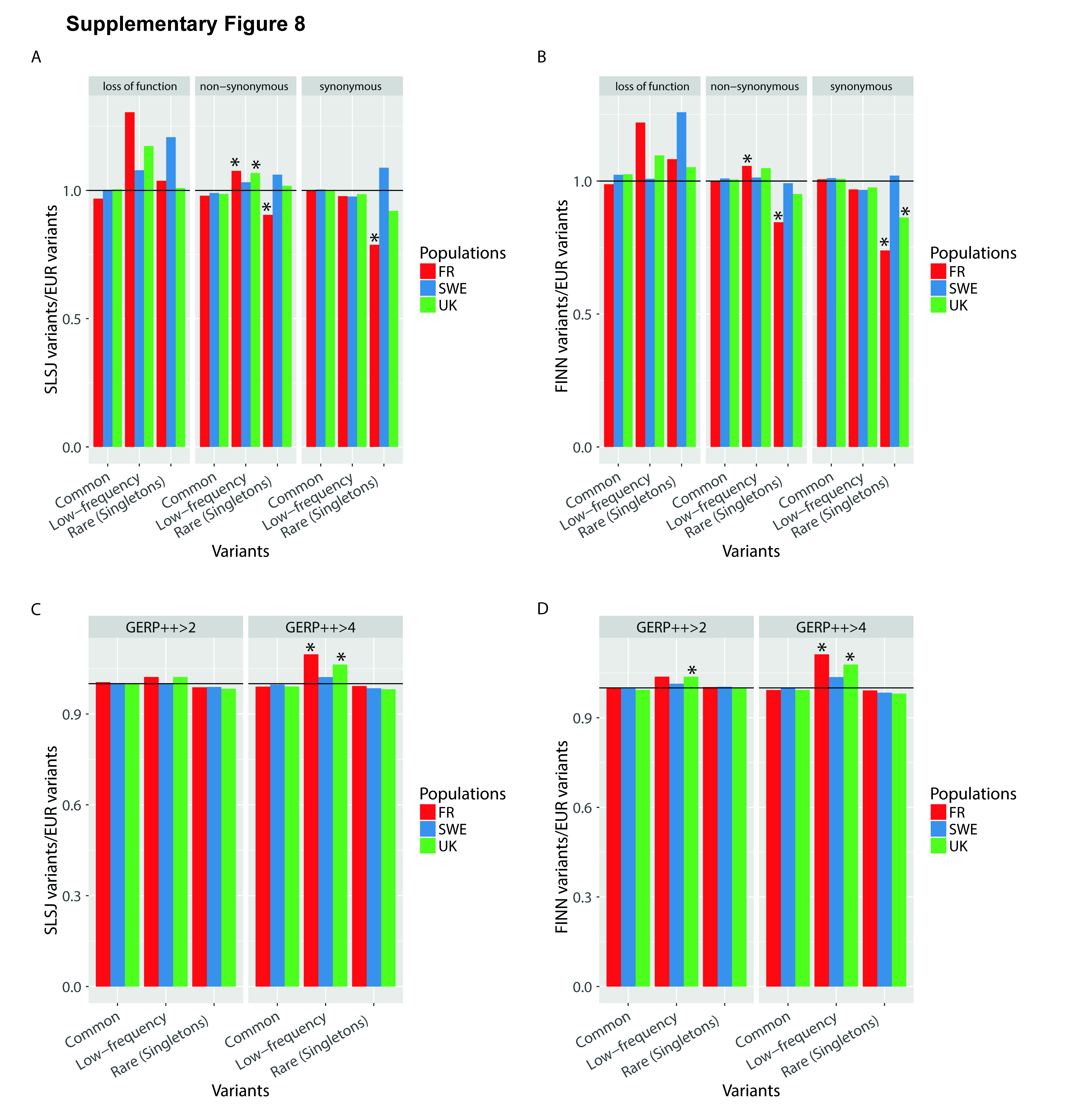

Supplement: Supplementary file 9 — Supplementary Figure 8. Common, low-frequency and singleton variants enrichment for deleterious variants [file 41431_2018_266_MOESM9_ESM.tif]

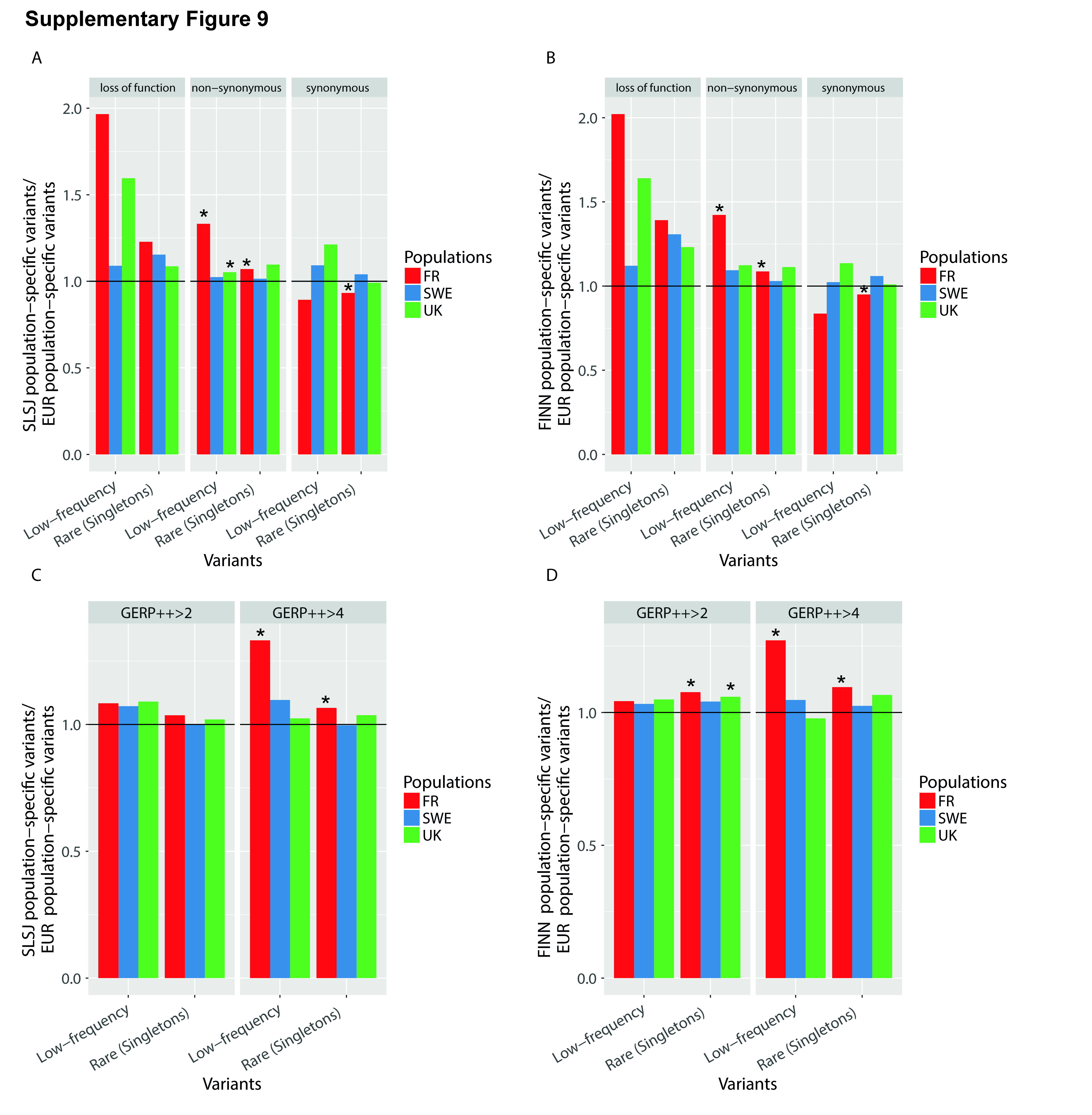

Supplement: Supplementary file 10 — Supplementary Figure 9. Population-specific low-frequency and singleton variants enrichment for deleterious variants [file 41431_2018_266_MOESM10_ESM.tif]

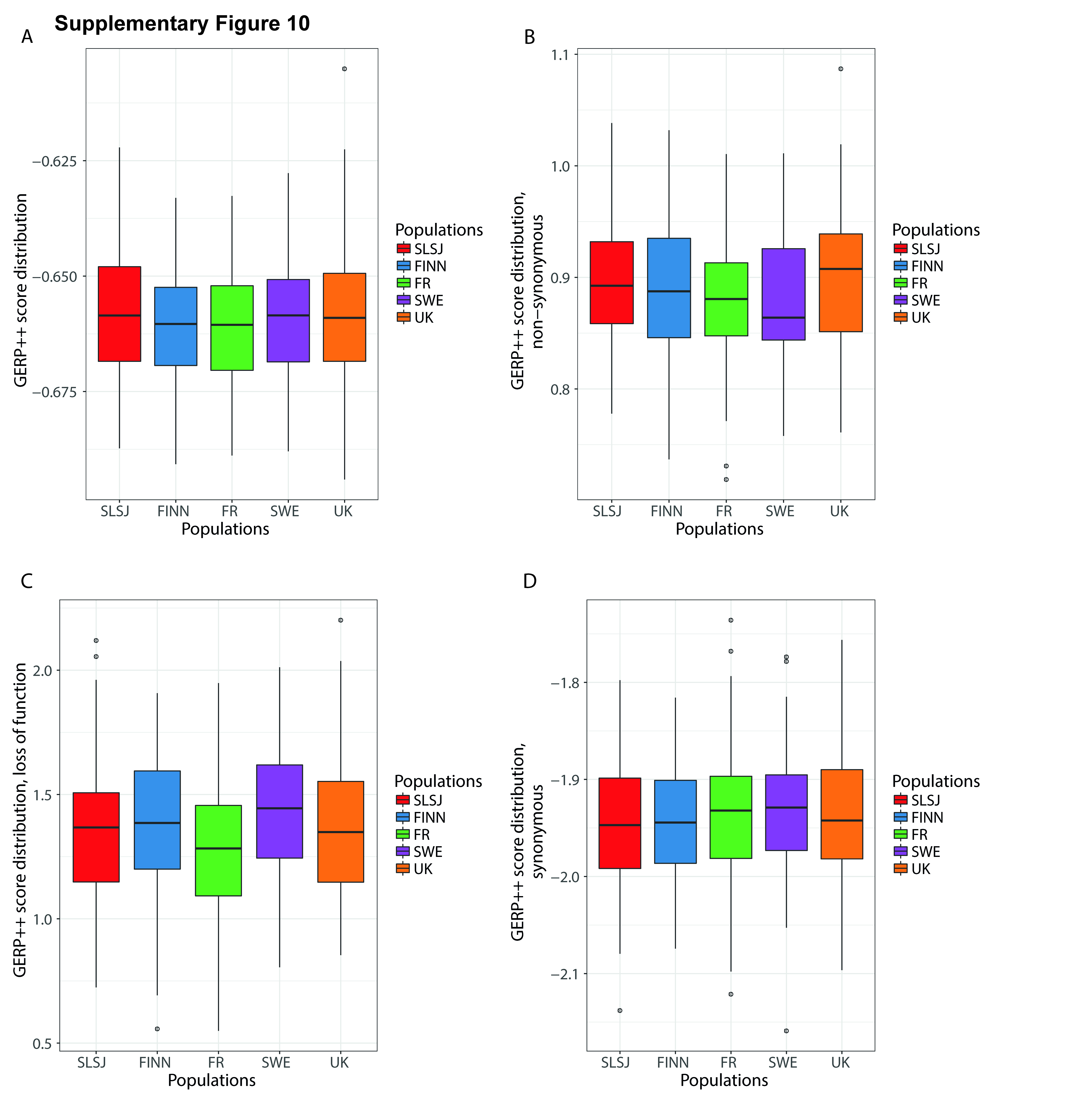

Supplement: Supplementary file 11 — Supplementary Figure 10. Average GERP++ per sample distribution [file 41431_2018_266_MOESM11_ESM.tif]

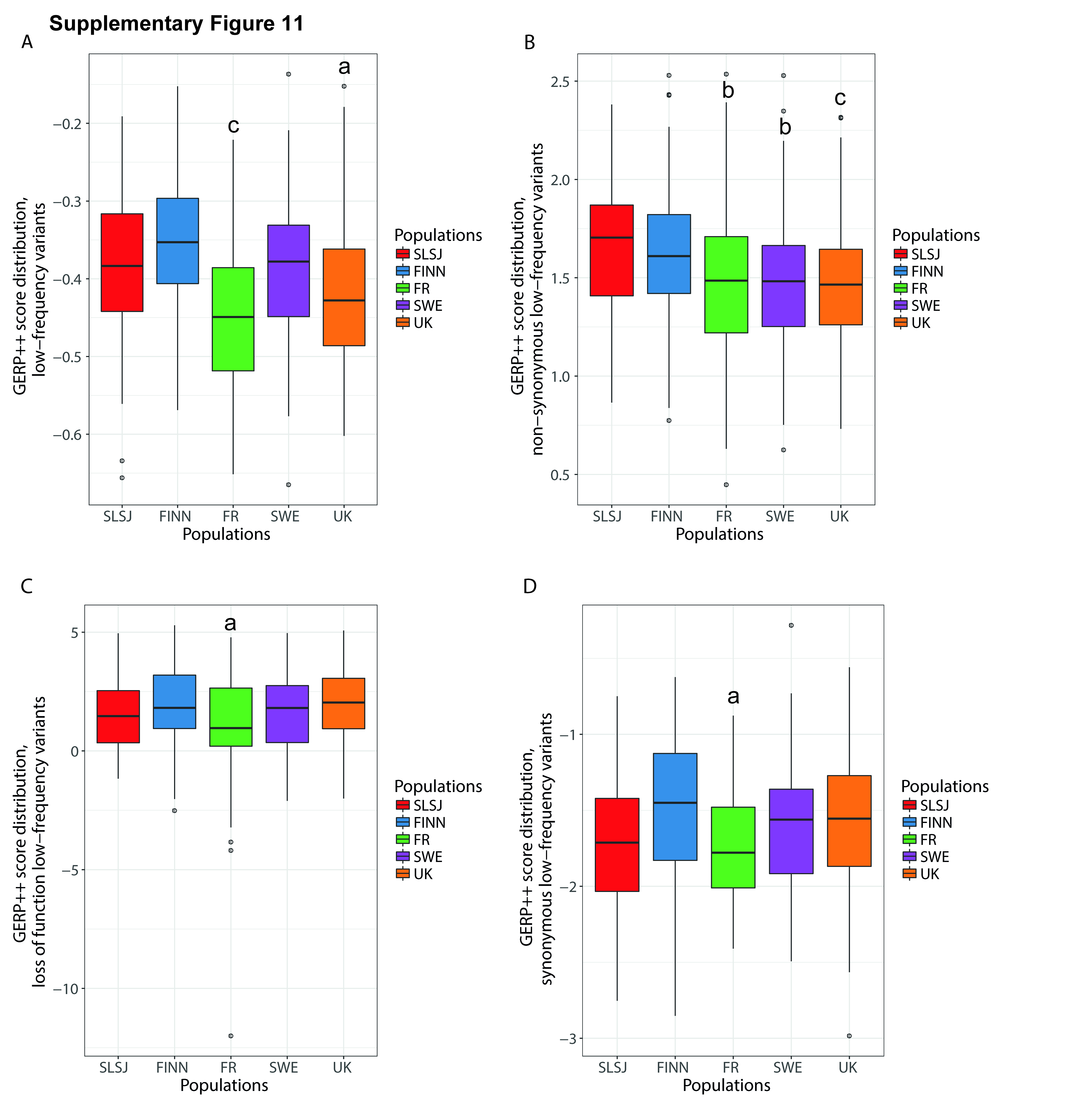

Supplement: Supplementary file 12 — Supplementary Figure 11. Average GERP++ per sample of low-frequency variants [file 41431_2018_266_MOESM12_ESM.tif]

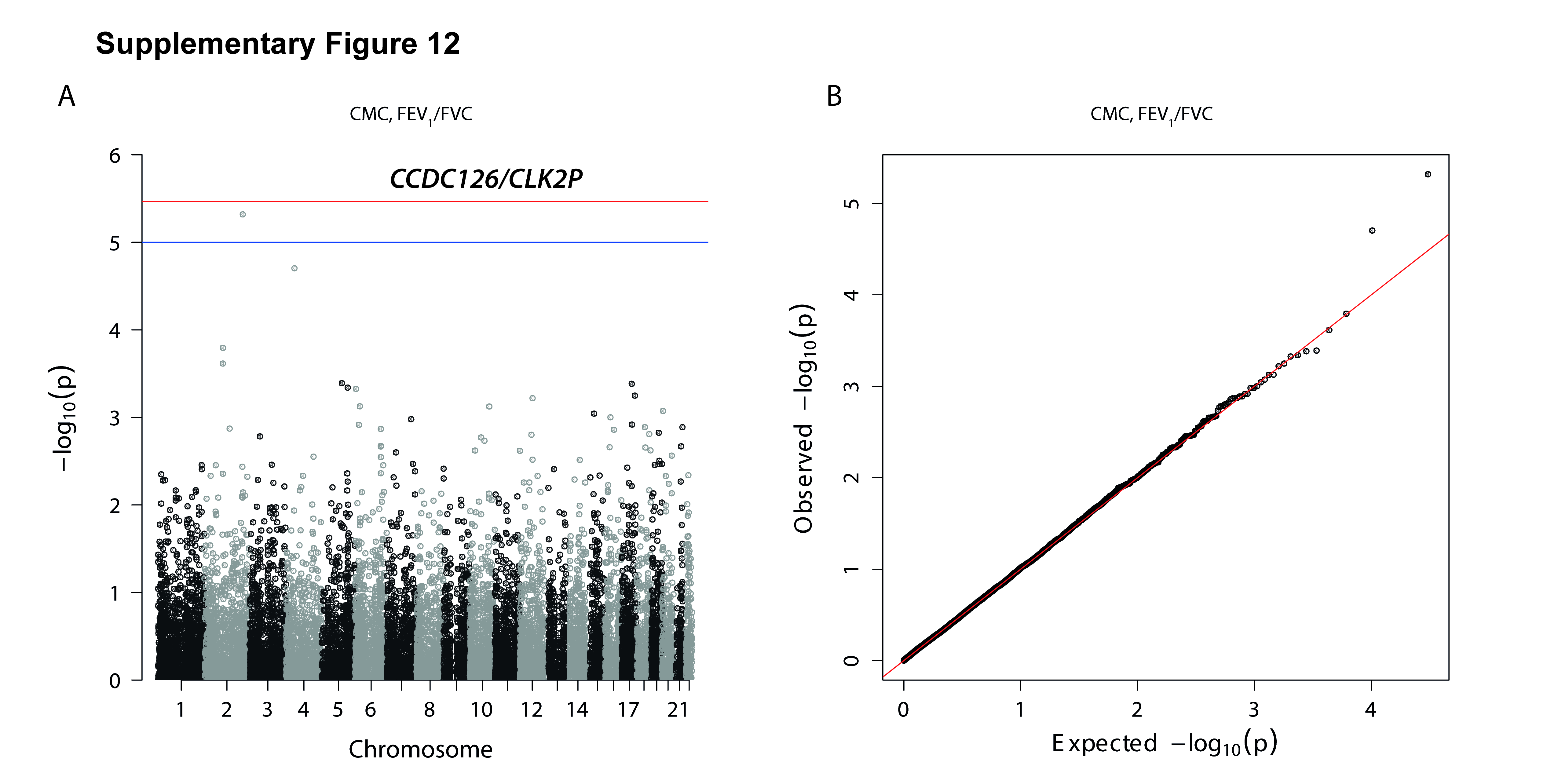

Supplement: Supplementary file 13 — Supplementary Figure 12. Manhattan plot and qqplot for CMC test with FEV1/FVC (Lambda= 1.04) [file 41431_2018_266_MOESM13_ESM.tif]
